# Oxygen glucose deprivation-pretreated astrocyte-derived exosomes attenuates intracerebral hemorrhage (ICH)-induced BBB disruption through miR-27a-3p /ARHGAP25/Wnt/β-catenin axis

**DOI:** 10.1186/s12987-024-00510-2

**Published:** 2024-01-19

**Authors:** Ying Hou, Ye Xie, Xiaoxuan Liu, Yushan Chen, Fangfang Zhou, Binbin Yang

**Affiliations:** Department of Neurology, 2nd Xiangya Hospital, Central South University, No. 139, Middle Renmin Road, Changsha, Hunan China

**Keywords:** Intracerebral hemorrhage, OGD pretreated astrocyte-derived exosome, Blood brain barrier, miR-27a-3p

## Abstract

**Background:**

Blood brain barrier (BBB) breakdown is one of the key mechanisms of secondary brain injury following intracerebral hemorrhage (ICH). Astrocytes interact with endothelial and regulate BBB integrity via paracrine signaling factors. More and more studies reveal astrocyte-derived extracellular vesicles (ADEVs) as an important way of intercellular communication. However, the role of ADEV in BBB integrity after ICH remains unclear.

**Methods:**

ADEVs were obtained from astrocytes with or without oxygen and glucose deprivation (OGD) pre-stimulation and the role of ADEVs in ICH was investigated using ICH mice model and ICH cell model. The potential regulatory effect of ADEVs on endothelial barrier integrity was identified by TEER, western blot and immunofluorescence in vitro. In vivo, functional evaluation, Evans-blue leakage and tight junction proteins (TJPs) expression were analyzed. MiRNA sequencing revealed that microRNA-27a-3p (miR-27a-3p) was differentially expressed miRNA in the EVs from OGD-pretreated astrocytes compared with normal control. The regulatory mechanism of miR-27a-3p was assessed using Luciferase assay, RT-PCR, western blot and immunofluorescence.

**Results:**

OGD-activated astrocytes reduced hemin-induced endothelial hyper-permeability through secreting EVs. OGD-activated ADEVs alleviated BBB dysfunction after ICH in vivo and in vitro. MicroRNA microarray analysis indicated that miR-27a-3p is a major component that was highly expressed miRNA in OGD pretreated-ADEVs. OGD-ADEVs mitigated BBB injury through transferring miR-27a-3p into bEnd.3 cells and regulating ARHGAP25/Wnt/β-catenin pathway.

**Conclusion:**

Taken together, these findings firstly revealed that miR-27a-3p, as one of the main components of OGD-pretreated ADEVs, attenuated BBB destruction and improved neurological deficits following ICH by regulating endothelial ARHGAP25/Wnt/β-catenin axis. OGD-ADEVs might be a novel strategy for the treatment of ICH.

this study implicates that EVs from OGD pre-stimulated astrocytes.

**Graphical Abstract:**

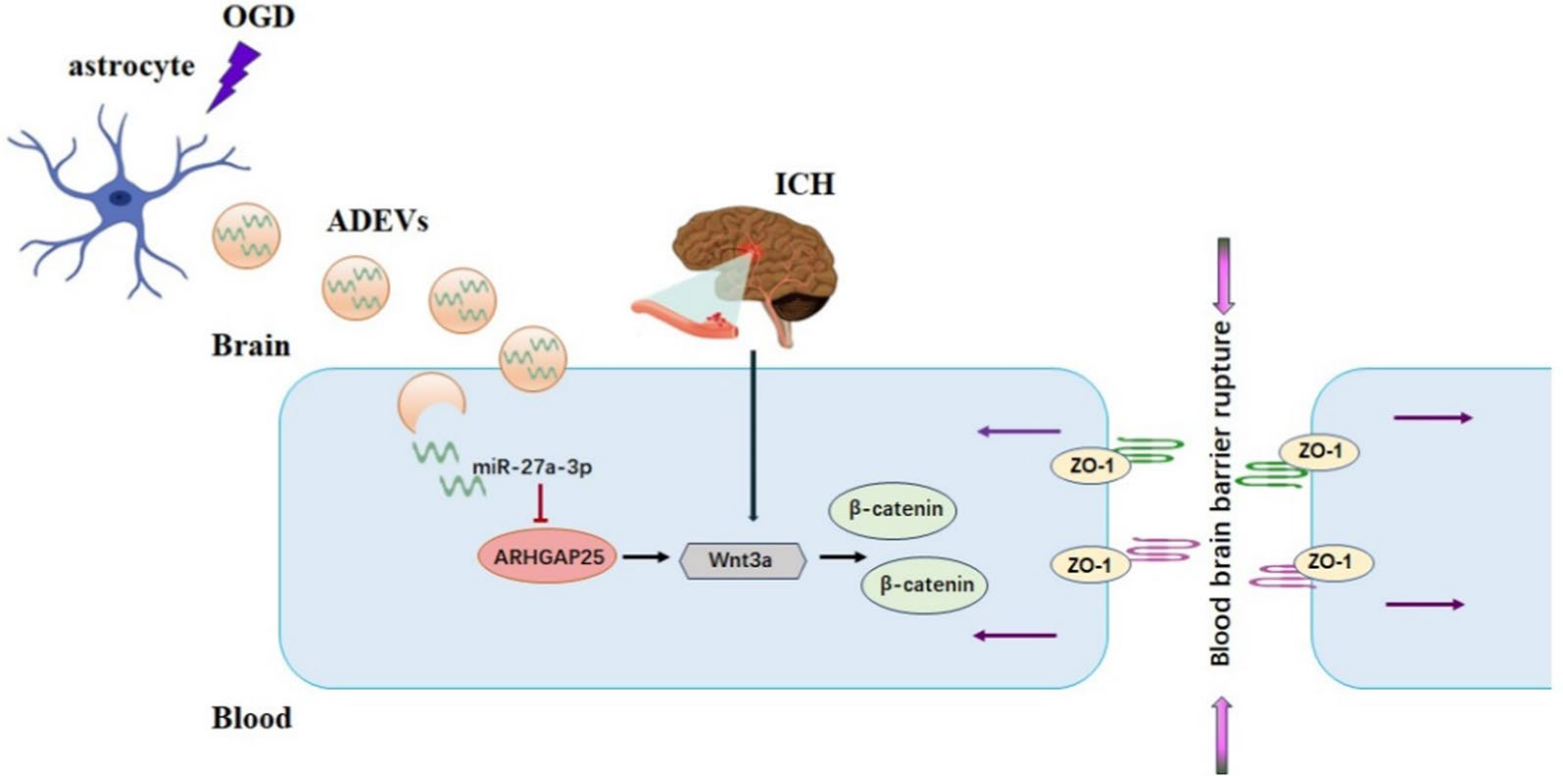

**Supplementary Information:**

The online version contains supplementary material available at 10.1186/s12987-024-00510-2.

## Background

ICH, which accounts for approximately 15% of strokes, is a devastating stroke type [1]. At least 30% of patients die within first month and 70% survivors have functional and cognitive impairment [2, 3]. Despite many pre-clinical studies and clinical trials have been performed recent years, effective therapeutic treatments to improve the prognosis of ICH patients are not available. Accumulating evidence indicates that a series of catastrophic effect caused by BBB disruption account for the poor prognosis of patients with ICH [4]. Following ICH, degradation of tight junction proteins (TJPs) contributes to BBB impairment, which subsequently leads to vasogenic edema formation, hemorrhagic transformation, and irreversible progressive deterioration [5, 6]. Therefore, BBB protection and recovery may represent a potential treatment strategy for ICH.

Astrocytes, brain microvascular endothelium and pericytes communicate with each other to synergistically produce signals required to maintain BBB integrity [7]. In recent years, it is increasingly clear that astrocytes play active roles in health and disease, and many crucial functions are executed in a paracrine manner [8–10]. ADEVs, as a fundamental component of astrocyte biology, have been attributed with pivotal roles in the central nervous system. Normal/resting astrocytes release a large number of EVs and perform homeostatic functions critical for the proper functioning of the CNS [11–13]. Under stimulation in pathological or aging conditions, astrocytes become alternatively activated, and cargo composition of ADEVs can be altered [14, 15]. The diversity in cargo allows ADEVs contribute to damage or repair in CNS diseases [16, 17]. Increasing evidence has suggested that astrocytes communicate with endothelial cells by secreting EVs and support BBB integrity [18]. However, studies focusing on the impact of ADEVs on BBB in ICH is still in its infancy.

The present study was implemented to evaluate, both in vivo and in vitro, the therapeutic effects of ADEVs on ICH-induced BBB injury. ADEVs, especially OGD-stimulated ADEVs (OGD-ADEVs), improved neurological function and alleviated BBB breakdown in an ICH mice model, and hindered hemin-induced endothelial barrier dysfunction in an ICH cell model. Using a bioinformatic analysis, the miRNAs enriched in OGD-ADEVs were sequenced to identify the key molecules specifically modified by hypoxia. Among top 10 upregulated miRNAs, miR-27a-3p was previously shown to modulate BBB during ICH. We further demonstrated that ADEVs transferred miR-27a-3p into bEnd.3 cells, which then targeted Rho GTPase Activating Protein 25(ARHGAP25)/Wnt/β-catenin axis and consequently protected against ICH-induced BBB injury.

## Methods

### Cell culture

Primary astrocytes were obtained and purified as previously described [19]. Briefly, brain cortex of neonatal (1–2 days old) C57/BL6J mice were collected, cut into pieces and were minced enzymatically (0.25% trypsin and 50 mg/mL of DNase I, 37 °C, 15 min). Cells were collected by mechanical trituration followed by centrifugation at 1500 rpm × 10 min. Cells were then re-suspended and seeded onto ploy-L-lysine 75-cm2 flasks, and incubated in the incubator (37 °C, 5% CO_2_). After 7–9 days, cell cultures were shaken at 300 rpm for 24 h at 37 °C to remove the loosely attached contaminated microglia and OPCs. Immunostaining confirmed astrocyte purity. Initially, the astrocytes were cultured in a base medium with an astrocyte growth supplement and fetal bovine serum. After 72 h of culture, astrocytes cells were treated with or without OGD for 6 h and then cultured in an EV-free medium consisting of the base medium plus EV-depleted serum and supplement in the 5% CO_2_ incubator at 37 °C for an additional 24 h. Afterwards, conditioned media (C.M.) were collected.

### In vitro* ICH model and monolayer BBB model*

The mouse brain endothelial cell line bEnd.3(ATCC) were seeded on a type I collagen-coated 12 mm transwell filters (1 × 105 cells/insert, 0.4 μm pore size; Corning Life Sciences) for 48 h until a confluent monolayer was established. In the subsequent experiments, treatment with 50 μM hemin for 24 h was selected as an in vitro model of ICH, according to previous study [20]. bEnd.3 cells were allocated into following groups: blank group (bEnd.3 cells received no treatment), hemin group(bEnd.3 cells treated with hemin), hemin + N-astrocyte group (bEnd.3 cells treated with hemin with half of cell medium replaced by the C.M. from normoxia-pretreated astrocytes), hemin + OGD-astrocyte group(bEnd.3 cells treated with hemin with half of cell medium replaced by the C.M. from OGD-pretreated astrocytes), hemin + OGD-astrocyte + GW4869 group(GW4869 at a concentration of 10 μM was added into astrocyte medium and cultured for 6 h. Then the medium was collected and cultured with bEnd.3 cells during hemin treatment); hemin + N-ADEVs group (bEnd.3 cells cocultured with EVs released normoxia-pretreated astrocytes 24 h before hemin treatment), hemin + OGD-ADEVs group (bEnd.3 cells cocultured with EVs released OGD-pretreated astrocytes 24 h before hemin treatment), hemin + OGD-ADEVs ^miR−27a−3p inhibitor^ group (bEnd.3 cells cocultured withOGD-ADEVs transfected with miR27a-3p inhibitor 24 h before hemin treatment), hemin + OGD-ADEVs^NC inhibitor^ group (bEnd.3 cells cocultured with OGD-ADEVs transfected with NC inhibitor 24 h before hemin treatment), hemin + miR27a-3p mimic + NC group (bEnd.3 cells co-transfected with miR-27a-3p mimic and NC vector 72 h before hemin treatment), hemin + miR27a-3p mimic + ARHGAP25 group (bEnd.3 cells transfected with miR-27a-3p mimic and ARHGAP25 overexpression pasmid simultaneously 72 h before hemin treatment). ADEVs were added to the culture medium of bEnd.3 cells in concentration of 20 μg/mL. The experiment’s schematic diagram for all experiments is shown in Additional file 1: Figure S1.

### *Evaluation of BBB integrity *in vitro

The integrity of monolayer bEnd.3 cells was evaluated by transepithelial electrical resistance (TEER). bEnd.3 cells were plated on the top of Transwell chambers and the TEER was monitored using an EndOhm Chamber connected to an EVOM2 epithelial voltmeter (World Precision Instrument), according to the instructions of the manufacturer. TEER value of the cell layers was obtained through sample TEER subtracting the background TEER values (the resistance of bank filters). TEER monolayer (Ω cm^2^) = [TEER total (Ω) − TEER blank (Ω)] × 4.67 (cm^2^).

### Separation and identification of astrocyte derived-EVs

ADEVs were isolated from the primary astrocyte supernatants as previously described [21]. Conditioned media were collected from 80 to 90% confluent astrocytes and centrifuged at 1500 ×*g* for 10 min. Supernatants were collected and sequentially centrifuged at 1000 × g for 10 min and 10,000 ×*g* for 30 min, followed by filtration using 0.22 μm needle filters. ADEVs were pelleted by centrifugation at 200,000 ×*g* for 2 h. Morphology of ADEVs was observed under transmission electron microscope (TEM), and quantified by Nanosight tracking analysis(NTA). The exosome protein content was measured using BCA protein assay (Thermo Scientific). Primary antibody of CD63 (ab216130, 1: 2000), TSG101 (ab125011, 1: 1000), Syntenin 1 (ab22595, 1: 100), and CD9 (ab92726, 1: 2000) were used to identify exosomes.

### *Cellular uptake of ADEVs *in vitro

ADEVs were stained with a green fluorescent dye PKH67 (Sigma Alderich, USA) according to the manufacturer's manual. bEnd.3 cells were incubated with stained ADEVs at 37 °C for 4 h to allow bEnd.3 cells to take up the ADEVs. After incubation, cells were washed twice with PBS and fixed with 4% paraformaldehyde for 15 min at 4 °C. The nuclei were stained with DAPI. Photographs were taken with a fluorescence microscope.

### ICH model

All animal experiments complied with the ARRIVE guidelines and and should be carried out in accordance with the National Research Council's Guide for the Care and Use of Laboratory Animals. All animal experiments were approved by the Ethics Committee of the 2nd Xiangya Hospital of Central South University. Adult male C57B/6 mice were 8–9 weeks of age. The ICH model was induced in a stereotaxic frame, according to previous report [22]. A 0.6 mm burr hole was drilled in the skull, and a 26-gauge needle was inserted through the burr hole into the left striatum (coordinates: 0.8 mm anterior, 3.0 mm ventral, and 2.0 mm lateral to the bregma). Collagenase IV (0.05 units in 0.5 μl of saline) was infused using a microinfusion pump at rate of 0.05 μl/ min. Animals were randomly assigned to four groups: sham group, ICH group, ICH + N-ADEVs group and ICH + OGD-ADEVs group. ADEVs were resuspended in 0.9% saline and injected intravenously through the tail vein at a concentration of 80 μg per 2 ml at 4 h after ICH induction, according to previous study [23].

### Behavioral tests

A modified Neurological Severity Scores (mNSS) was employed to evaluate the neurological function at 1, 3 and 7 days after ICH, as previously described.

### Brain water content assessment and evans blue extravasation assay

The brains were removed and hemispheres were separated and weighed immediately (wet weight). The hemispheres were weighed again after dried for 24 h at 110 °C. Brain water content was assessed as: (wet weight-dry weight)/(wet weight) × 100%.

BBB permeability was investigated by the leakage of Evans Blue (EB) extravasation. 2% EB dye (2 mL/kg) was injected through the tail vein followed by transcardially perfusion with PBS. Both the left and right hemisphere tissues of the brain were then collected and weighed, soaked in formamide solution for 24 h at 55 °C. Samples were centrifuged at1200 ×*g* for 20 min at 4 °C. The supernatants collected and EB content was measured with a spectrophotometer (λ = 632 nm) and was calculated using a standard curve.

### Exosomal miRNA sequencing

Total RNA was extracted from N-ADEVs and OGD-ADEVs using miRNeasy Micro Kit (QIAGEN) according to the manufacture’s protocol. The libraries of small RNAs were sequenced using Illumina HiSeqTM 2500. Significance of differentially-expressed miRNAs between two groups were identified through fold change > 1.5. P value < 0.05 was set as the threshold. Target gene prediction, target gene functional enrichment and Kyoto Encyclopedia of Genes and Genomes (KEGG) enrichment analysis were also performed.

### Detection of Cy3-labelled miR-27a-3p transfer

Astrocyte was transfected with Cy3 labeled miR-27a-3p mimic. ADEVs extracted by the above method of separation and purification of exosomes. The ADEVs, including Cy3- miR-27a-3p, were stained with PKH67 according to the manufacturer’s instructions. For fluorescence microscopy analysis, bEnd.3 cells were seeded on 24-well plate cell slides in a 5% CO2 incubator for 24 h and co-incubated with stained exosomes for 6 h. Cells were fixed with 4% PFA and then stained with DAPI. The slides were visualized under a confocal fluorescence microscope.

### Cell transfection

The miR-27a-3p inhibitor, corresponding negative controls (inhibitor NC), were purchased from GenePharma (Shanghai, China). miR-27a-3p inhibitor or inhibitor NC were transfected into astrocytes using Lipofectamine^®^ 2000 (Invitrogen, USA), according to the manufacturer’s protocols. EVs extracted from mi-R transfected astrocytes were OGD-ADEVs ^miR−27a−3p inhibitor^ or OGD-ADEVs ^NC inhibitor^ and used for the treatment of BMECs.

### Reverse transcription-quantitative polymerase chain reaction (RT-qPCR)

Total RNA was isolated using Trizol reagent (Invitrogen) and reverse-transcribed to cDNA using using an RT2 First Strand Kit (Qiagen). qRT-PCR was conducted using the SYBR Premix ExTaq II. The relative expression of miRNA expression was evaluated using the –2^ΔΔCt^ method. The primer sequences were shown in Table 1.

**Table 1:** The primer sequences used for Real-Time PCR

miR-27a-3p	Sense	5ʹ—TGAGGAGCAGGGCTTAGCTG—3ʹ
	Antisense	5ʹ—AACCACCACAGATTCACTAT—3ʹ
GAPDH	Sense	5ʹ—GCGGAACTTAGCCACTGTGA—3
	Antisense	5ʹ—GCAGGAGGCATTGCTGAT—3ʹ

### Western blot

Total protein was isolated from brain samples and bEnd.3 cells was lysed with RIPA lysis buffer. The protein concentration was determined by the BCA kit. Next, the same amounts of proteins from each sample were electrophoresed on a 12% SDS-PAGE gels and then transferred to the PVDF membrane. The membranes were blocked with 5% nonfat skim milk for 2 h at room temperature and probed overnight at 4 °C with the primary antibody. HRP-labeled secondary antibody were used for detection on ChemiDoc XRS system (Bio-rad). The membranes were then incubated with HRP-conjugated secondary anti-rabbit antibody (1:2000, ab288151, Abcam) or mouse antibody (1:5000, ab97040, Abcam) for 1 h at room temperature, and then visualized with enhanced chemiluminescence (ECL) reagent on ChemiDoc XRS system (Bio-rad). β-actin was cited as the internal reference. Primary antibodies were as follows: occluding (1:1000, ab216327, Abcam), claudin-5(1:500, 35–2500, Invitrogen), ZO-1(1:1000, ab276131, Abcam), ARHGAP25 (1:1000, ab192020, Abcam), β-catenin (1:1000, 9562, Cell signaling), wnt3a (1:1000, ab219412, Abcam), β-actin (1:2000, ab210083, Abcam).

### Immunofluorescence staining

Coronal brain Sects. (20 μm thick) or bEnd.3 cells were blocked for 1 h with 5% goat serum. Sections were immunostained with primary antibodies against ZO-1 (5 μg/ml, 40–2300, Thermo Fisher Scientific) overnight at 4 °C overnight. After being washed with PBS, sections were incubated with corresponding secondary antibodies containing Alexa Fluor 647 Donkey Anti-Goat IgG or Alexa Fluor 488 donkey anti-mouse IgG at room temperature for 1 h. The images were captured by a fluorescent microscope and analyzed using ImageJ software.

### Dual-Luciferase reporter gene assay

ARHGAP25 3ʹ UTR sequence (ARHGAP25-WT) or the mutant sequence of ARHGAP25 3ʹ UTR containing the predicted binding sites of miR-27a-3p (ARHGAP25-MUT) was cloned into the pGL3 promoter vector. The luciferase reporter plasmids were transfected into 293 T cells, respectively and then with miR-27a-3p mimic or mimic NC. The relative activity of luciferase was detected by dual-luciferase reporter assay kit.

### Statistical analysis

The data are shown as the mean ± standard deviation. Two-group differences were analyzed by unpaired Student’s *t* test. Multiple-group differences were analyzed by one-way analysis of variance followed by Dunnett’s post hoc analysis. Statistical analyses were performed with GraphPad Prism software. Statistical significance was defined as *p* < 0.05.

## Results

### OGD-activated astrocytes reduced hemin-induced endothelial hyper-permeability through secreting EVs

**Fig. 1:**
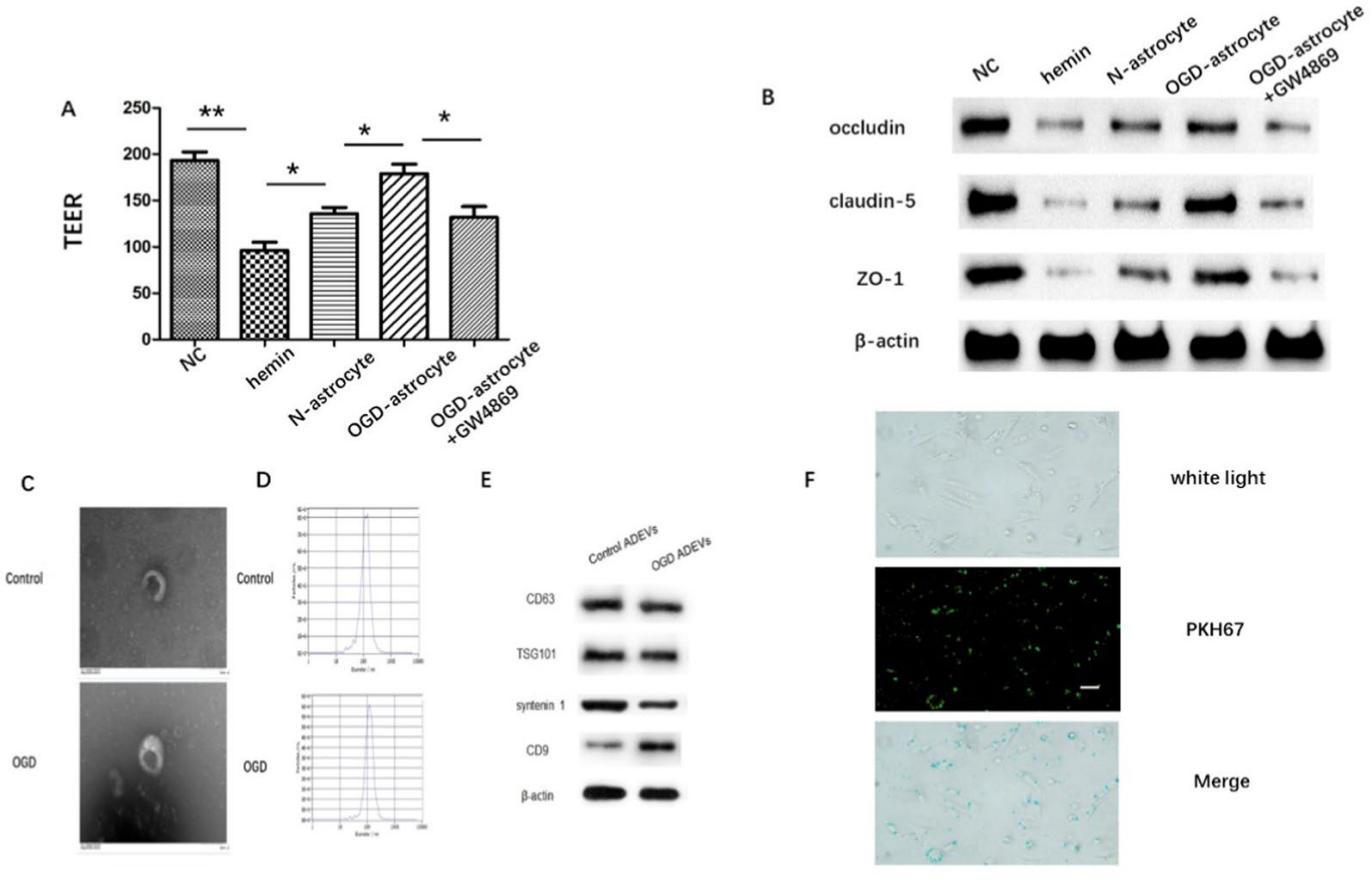
Astrocyte protected against hemin-induced BBB disruption through secreting exosomes **A**. TEER of monolayer bEnd.3 cells. **B**. Immunoblot images showing the expression of occludin, claudin-5 and ZO-1 in bEnd.3 cell monolayers. **C**. Morphological characterization of ADEVs observed under a TEM. **D**. The concentration and size distribution of ADEVs analyzed by NTA. E. Western blot analysis of EV surface maker proteins TSG101, CD81, CD63, and CD9. F. Fluorescence microscope was used to observe the uptake of PKH67-labeled ADEVs (green) by bEnd. 3 cells. Scale bar: 20 μm. **p < 0.01 versus indicated group; *p < 0.05 versus indicated group. All data were expressed as mean ± SEM of at least 3 independent experiments

We stimulated bEnd.3 cells with 50 μM hemin for 24 h to mimic ICH conditions in vitro. bEnd.3 cells were treated with conditioned medium (C.M.) from astrocytes with or without OGD pre-stimulation, respectively. The TEER of monolayer bEnd.3 cells was decreased significantly by hemin stimulation, while C.M. from astrocyte significantly refrained hemin-induced decrease of TEER (Fig. 1A). Notably, C.M. from OGD-preconditioned astrocytes (OGD-astrocytes) displayed.

showed stronger protective ability than that from normal astrocyte (N-astrocyte) (Fig. 1A). Studies have shown that disturbance of TJ proteins, mainly including claudin-5, ZO-1, and occludin, contributes to impairment of the BBB [24]. Western-blot in our study showed that hemin decreased the expression of tight junction proteins (TJPs), occludin, claudin-5 and ZO-1, which was partially restored by C.M. from astrocytes. OGD-astrocyte group had stronger rescuing capacity than N-astrocyte (Fig. 1B, Additional file 1: Figure S2). To verify the paracrine mechanism of astrocyte through EVs release, we inhibited exoxomal secretion with GW4869. The ability of astrocytes to restore barrier integrity was offset by the treatment with GW4869 (Fig. 1A–B). EVs were then isolated from astrocytes by ultracentrifugation and evaluated as described below. TEM showed that both normal and OGD-preconditioned astrocyte-derived vesicles were round-shaped nanovesicles (Fig. 1C).NTA revealed that both ADEVs had diameters ranging from 30 and 200 nm (Fig. 1D). Additionally, western blot analysis indicated that ADEVs were positive for surface markers (CD63, TSG101, syntenin 1 and CD9) of EVs (Fig. 1E). The data demonstrated the successful isolation of ADEVs. The extracted ADEVs were labeled via PKH67 green fluorescence and co-cultivated with bEnd.3 cells. After 6 h of culture, PKH67 green fluorescence was observed in bEnd.3 cells, indicating ADEVs were internalized by bEnd.3 cells (Fig. 1F).

### ADEVs alleviated BBB dysfunction after ICH in vivo and in vitro

**Fig. 2:**
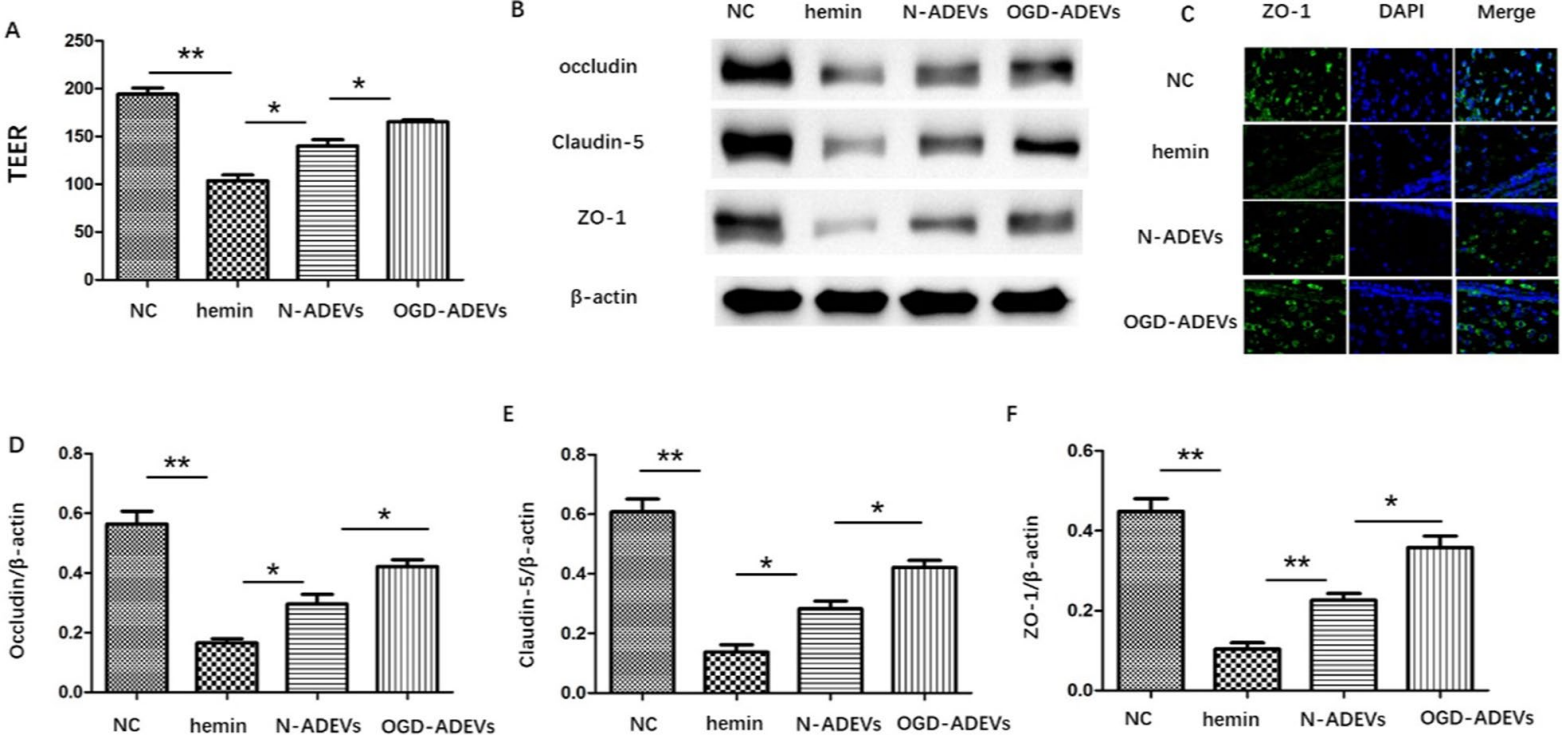
ADEVs protected against barrier hyperpermeability and TJPs disruption in bEnd.3 cells after hemin treatment **A**. Determination of TEER to evaluate the barrier integrity of monolayer bEnd.3 cells. **B**. Western blot analysis of tight junction protein markers (occludin, Claudin-5, and ZO-1) in bEnd.3 cells. **C**. Immunofluorescence staining for ZO-1 in bEnd.3 cells **D**–**E**. Quantification of the relative protein expression levels of occludin, Claudin-5, and ZO-1. **p < 0.01 versus indicated group; *p < 0.05 versus indicated group. All data were expressed as mean ± SEM of at least 3 independent experiments

To explore the effect of ADEVs on BBB integrity after ICH, the leakage of microvascular endothelial barrier was measured using TEER. TEER of the bEnd.3 cell monolayer models was destroyed by hemin. The reduction of TEER was significantly improved by N-ADEVs coculturing (vs. hemin group). A more significant increment was found after treating with OGD-ADEVs (Fig. 2A). The protein expression of claudin-5, ZO-1, and occludin was significantly decreased in hemin group, compared to NC, while ADEVs coculturing could partially reverse this decline. Compared with N-ADEVs, OGD-ADEVs showed a better improvement (Fig. 2B, D–F). The results of immunofluorescence staining were consistent with the above results. Compared with hemin group, the expression of ZO‐1 was significantly increased in N-ADEVs and OGD-ADEVs group. Specifically, the expression of ZO‐1 in OGD-ADEVs was higher than N-ADEVs (Fig. 2E).

**Fig. 3:**
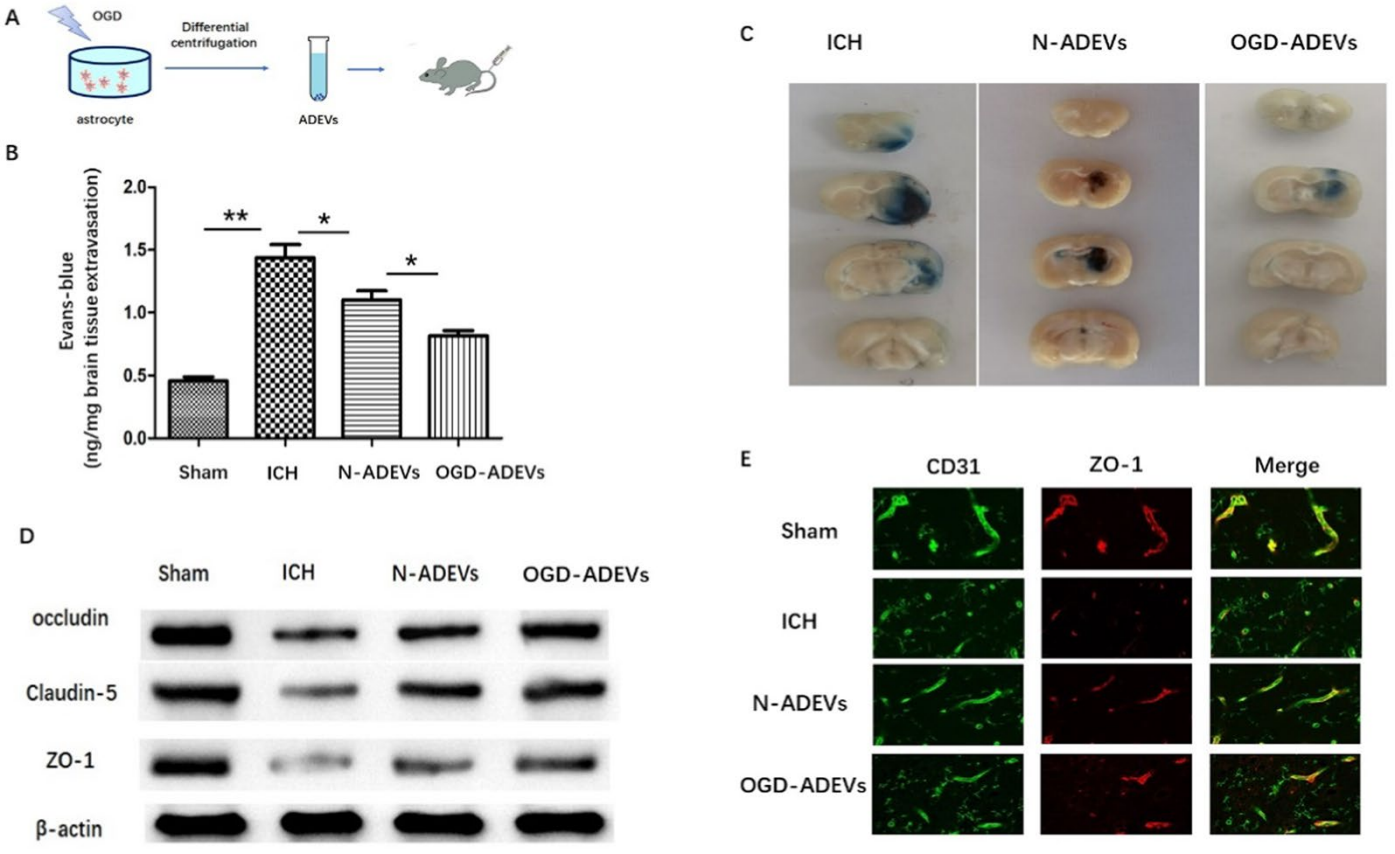
ADEVs mitigated ICH-induced BBB injury **A**. Schematic illustration presented that EVs from OGD-stimulated astrocytes was injected into mice from tail vein. **B**. Representative images showing EB extravasation 3 days after ICH induction. **C**. Quantitative analysis of EB extravasation. **D**. Representative western blot image of occludin, claudin‐5 and ZO-1 in peri-hematoma region of mice brain were measured by western blotting. **E**. Immunofluorescence staining for ZO-1/CD31 in the peri-hematoma region. Scale bar: 20 μm. **p < 0.01 versus indicated group; *p < 0.05 versus indicated group. All data were expressed as mean ± SEM of at least 3 independent experiments

We next examined the effect of ADEVs on BBB in ICH mice (Fig. 3A). The results of the neurological score are shown in Additional file 1: Figure S3. Compared with sham group, ICH mice exhibited higher neurological deficit scores on days 1, 3, and 7. ADEVs treatment led to improvement in the mNSS. Notably, compared with N-ADEVs, OGD-ADEVs treatment recovered neurological function to higher level. Brain water content was investigated on the 3rd-day post-ICH. ICH remarkably increased the brain water content in the ipsilateral brain. ADEVs treatment dramatically reduced brain water content (Additional file 1: Figure S3). Additionally, in the ICH group, EB extravasation increased remarkably compared with sham group. However, this leakage was attenuated by ADEVs administration. The degree of EB leakage in OGD-ADEVs group was relatively lower than that in N-ADEVs group (Fig. 3B–C, D) showed that ICH-indued downregulation of ZO-1, occludin, and claudin-5 level was greatly reversed by ADEVs administration. Notably, the TJP levels were strengthened in OGD-ADEVs group compared with N-ADEVs group with statistically significant differences (Fig. 3D, Additional file 1: Figure S4). Co-immunostaining of ZO-1 with CD31 showed linear distribution of ZO-1 in normal brains. After ICH, we observed disruption of junction arrangement, indicative of damaged BBB. However, in the N-ADEVs and OGD-ADEVs treatment group, the co-localization of ZO-1 and CD31 around the hematoma areas was markedly increased compared to ICH animals (Figs. 3E).

### miRNA expression profile of OGD-ADEVs

**Fig. 4:**
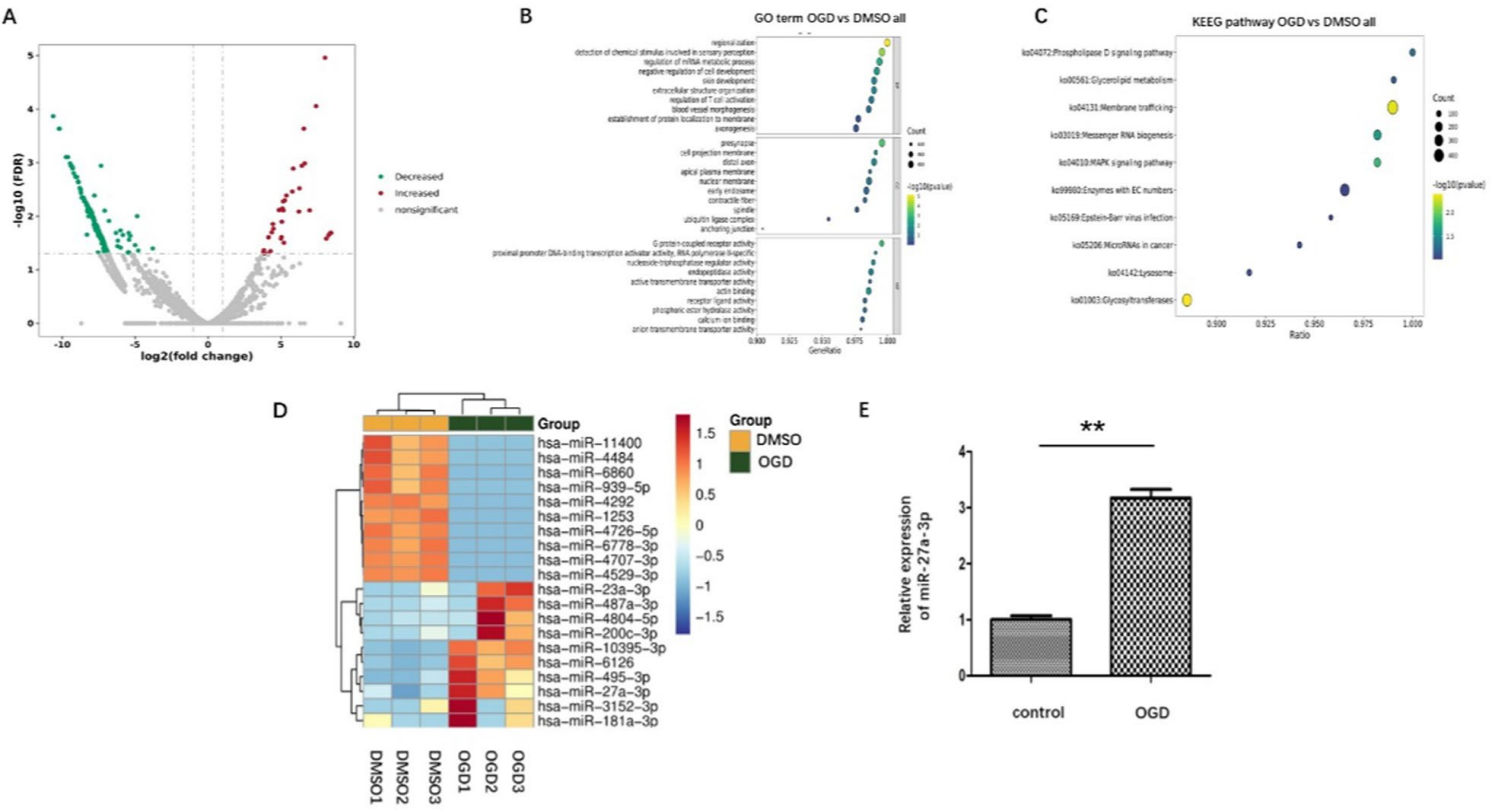
miRNA expression signature profiling in OGD-ADEVs **A**. miRNA volcano plot. Red point in the plot represents differentially upregulated miRNAs, and green point represents downregulated miRNAs, with statistical significance (fold change > 2.0 or < − 2.0; P < 0.05) **C**–**D**. The bubble map of GO enrichment and KEGG pathway analysis of miRNA target genes. **D**. A heat map of the first 20 significantly differentially expressed miRNAs. **E**. Expression of miR-27a-3p in N-ADEVs and OGD-ADEVs evaluated by qRT-PCR

An increasing number of studies has shown that EVs-contained miRNAs play protective roles in CNS diseases, including stroke [25, 26]. Hence, we focused our mechanistic investigation on ADEVs-contained miRNAs. To identify differentially expressed miRNAs in EVs released from hypoxic astrocyte, a high-throughput miRNA-sequencing was performed in our study. All produced reads were compared with the known human miRNAs in miRbase. A volcano plot of differentially enriched miRNAs showing 36 up-regulated miRNAs and 244 down-regulated miRNAs in the OGD-ADEVs compared with N-ADEVs [Log2(fold change) > 2] (Fig. 4A). Then, GO analysis of target genes (Fig. 4B), and KEGG analyses (Fig. 4C) were performed. The results showed that miR-27a-3p was one of the most abundant encapsulated signaling molecules in OGD-ADEVs (Fig. 4D), which has been reported to maintain BBB integrity [27]. To validate the elevated miR-27a-3p expression in OGD-ADEVs, qRT- PCR was performed. The results showed that miR-27a-3p was significantly elevated in OGD-ADEVs compared with N-ADEVs (Fig. 4E).

### OGD-ADEVs suppress hemin-induced barrier hyperpermeability through transferring miR-27a-3p

**Fig. 5:**
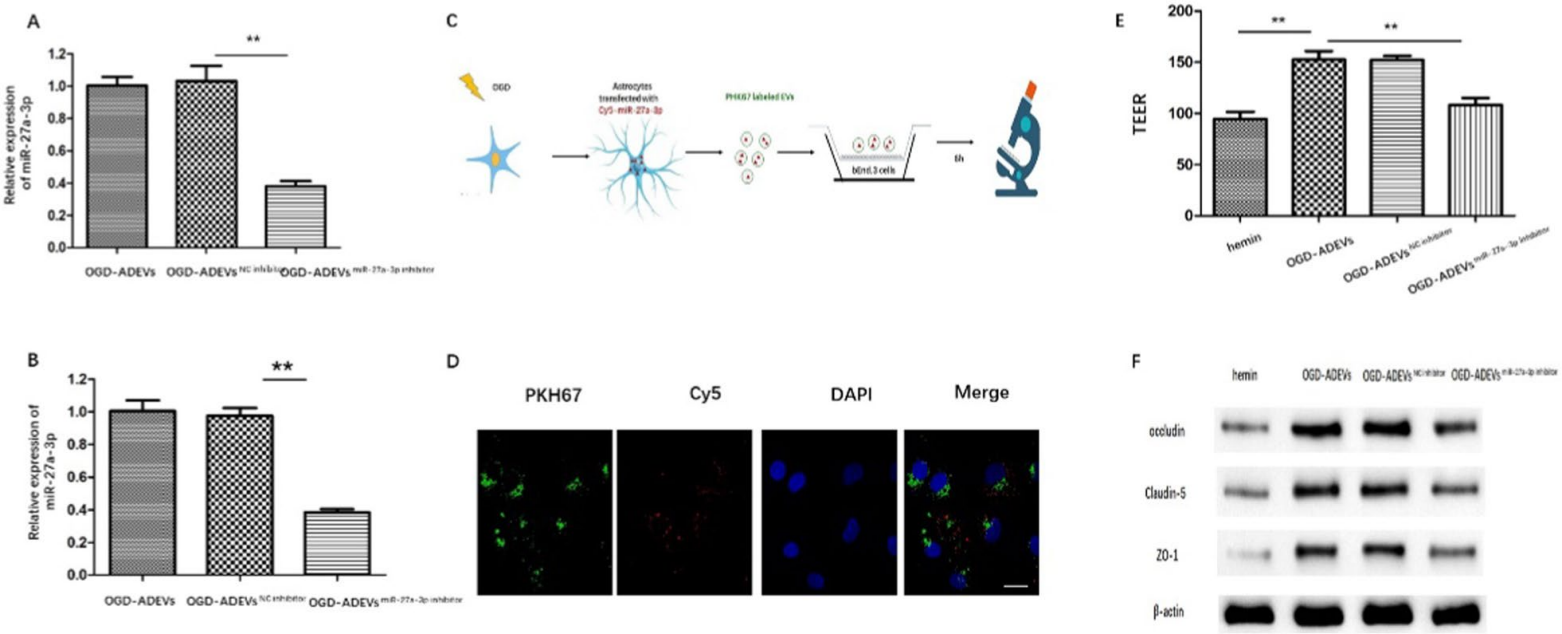
ADVEs abrogated hemin-induced barrier injury in bEnd.3 cells through transferring miR-27a-3p **A**. RT-qPCR analysis of miR-27a-3p expression in ADEVs from different groups. **B**. RT-qPCR analysis of miR-27a-3p expression in BMECs from different groups. **C**. Schematic illustration presented that OGD-stimulated astrocyte was transfected with Cy3 labeled miR-27a-3p mimic, ADEVs extracted was labeled with PKH67 and incubated with bEnd. 3 cells. **D**. Confocal images of PKH67- labeled ADEVs, including Cy3-miR-27a-3p, internalized by bEnd.3 cells. **E**. TEER of monolayer bEnd. 3 cells. Scale bar: 20 μm **F**. The protein levels of occludin, claudin-5 and claudin-5 in bEnd.3 cells determined by western blot. **p < 0.01 versus indicated group; *p < 0.05 versus indicated group. All data were expressed as mean ± SEM of at least 3 independent experiments

We next investigate whether miR-27a-3p can be transferred from astrocytes to bEnd.3 cells by ADEVs. We knocked down miR-27a-3p in OGD-ADEVs through miR-27a-3p inhibitor transfection. The expression of miR-27a-3p in OGD-ADEVs was detected by RT-qPCR. miR-27a-3p expression was decreased in OGD-ADEVs ^miR−27a−3p inhibitor^ compared with OGD-ADEVs and OGD-ADEVs ^NC inhibitor^ (Fig. 5A). bEnd.3 cells were incubated with untransfected OGD-ADEVs, OGD-ADEVs ^miR−27a−3p inhibitor^ or OGD-ADEVs^NC inhibitor^ for 6 h, respectively. qRT-PCR analysis showed that miR-27a-3p was significantly downregulated in bEnd.3 cells incubated with OGD-ADEVs^miR−27a−3p inhibitor^ compared with OGD-ADEVs and OGD-ADEVs ^NC inhibitor^ (Fig. 5B). Furthermore, OGD-ADEVs were labelled with PKH67 (green) and then transfected with Cy3 labeled miR-27a-3p mimic (red). Labeled ADEVs were incubated with bEnd.3 cells for 6 h (Fig. 5C). As shown in Fig. 5D, PKH67-labeled OGD-ADEVs entered bEnd.3 cells. In addition, Cy3-labeled miR-27a-3p mimic were found inside bEnd.3 cells. Collectively, these data indicated that miR-27a-3p can be transferred from hypoxic astrocytes to endothelia via the route of EVs.

To explore whether OGD-ADEVs-enriched miR-27a-3p is involved in the regulation of TJs after ICH in vitro, hemin-stimulated bEnd.3 cells were incubated separately with OGD-ADEVs, OGD-ADEVs ^NC inhibitor^, and OGD-ADEVs ^miR−27a−3p inhibitor^. Hemin stimulation markedly decreased the TEER of monolayer of bEnd.3 cells, whereas the decrease was mitigated by OGD-ADEVs or OGD-ADEVs ^NC inhibitor^ administration. The effect of OGD-ADEVs was reversed by miR-27a-3p inhibitor. The western blot analysis showed that OGD-ADEVs or significantly reduced the expression of occludin, claudin-5 and ZO-1. However, the effect disappeared after transfected with miR-27a-3p inhibitor (Fig. 5F, Additional file 1: Figure S5). These results revealed OGD-ADEVs attenuated hemin-induced BBB injury, at least, partially through miR-27a-3p.

### ARHGAP25 is a direct target of miR-27a-3p

**Fig. 6:**
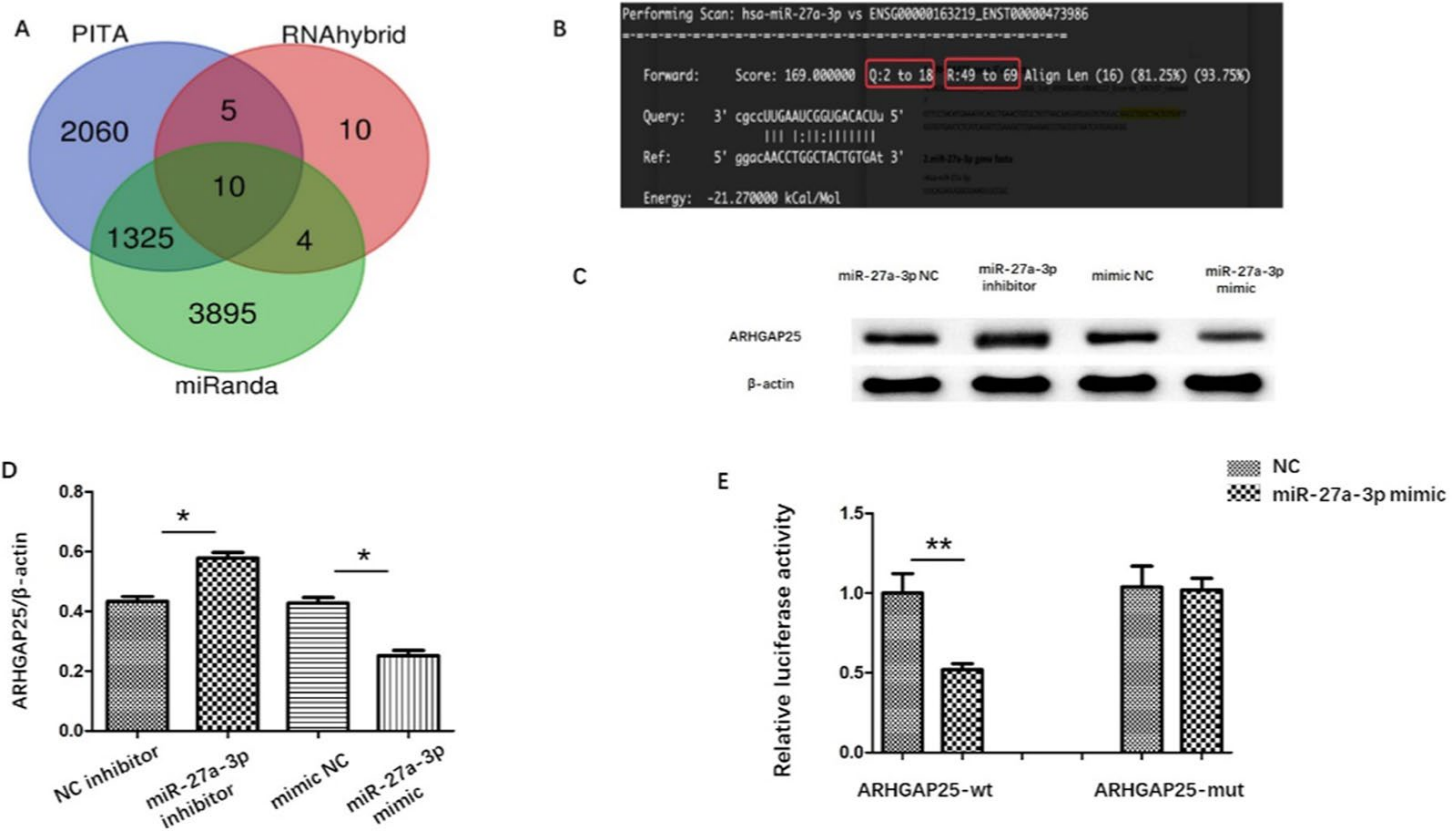
ARHGAP25 was a target gene of miR-27a-3p **A**. Venn diagram analysis of the predicted downstream target genes of miR-27a-3p by miRanda, PITA and RNAhybrid databases. **B**. The putative miR-27a-3p binding sites in ARHGAP25 C-D. Western blot and quantitative analysis of ARHGAP25. **E**. Luciferase reporter assay to evaluate the interaction ability between miR-27a-3p 3ʹ—UTR and ARHGAP25. **p < 0.01 versus indicated group; *p < 0.05 versus indicated group. All data were expressed as mean ± SEM of at least 3 independent experiments

We next probed the molecular mechanisms underlying the potential role of OGD-ADEV-enriched miR-27a-3p in BBB protection. Target genes of miR-27a-3p were predicted using the miRanda, PITA and RNAhybrid databases (Fig. 6A). Through the miRanda database, Rho GTPase activating protein 25(ARHGAP25) as a target gene of miR-27a-3p was found. The binding sequence of miR-27a-3p and ARHGAP25 was shown in Fig. 6B. We transfected miR-27a-3p inhibitor in bEnd.3 cells and detected ARHGAP25 expression. miR-27a-3p inhibitor caused ARHGAP25 upregulation. Conversely, miR-27a-3p mimic caused downregulated ARHGAP25 expression (Fig. 6C–D). A subsequent luciferase reporter assay indicated that miR-27a-3p mimic decreased luciferase intensity in HEK293T cells transfected with entire WT‐ARHGAP25 3'UTR but not MUT ARHGAP25 3′UTR (Fig. 6E). These data indicated that miR-27a-3p could directly target ARHGAP25.

### miR-27a-3p attenuates BBB disruption via regulating ARHGAP25/Wnt/β-catenin pathway

**Fig. 7:**
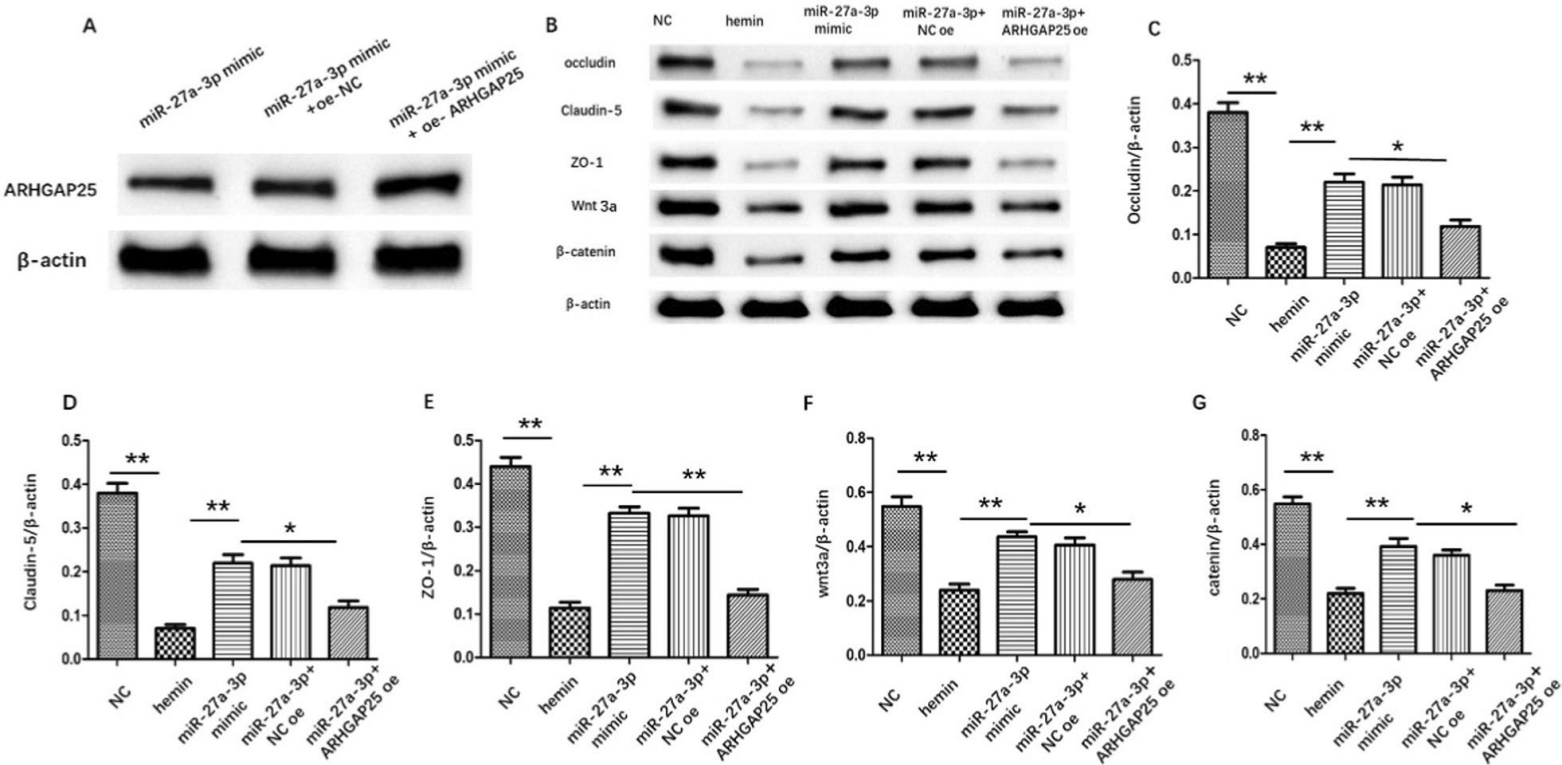
miR-27a-3p suppressed TJPs degradation via ARHGAP25/Wnt/β-catenin pathway **A**. Western blot analysis of ARHGAP25 **B**. Protein expression of occludin, claudin‐5, ZO-1, Wnt3a and β-catenin by western-blot. **C**–**G**. Quantitation of the bands in B. **p < 0.01 versus indicated group; *p < 0.05 versus indicated group. All data were expressed as mean ± SEM of at least 3 independent experiments

Western blot showed that expression of ARHGAP25 was downregulated by miR-27a-3p mimic transfection, and co-transfection with miR-152-3p mimic and oe-ARHGAP25 reversed the reduction of ARHGAP25 expression (Fig. 7A). As illustrated in Fig. 7B–E, overexpression of miR-27a-3p increased the occludin, claudin-5 and ZO-1 compared with hemin group, while the effect was inhibited by co-transfection with miR-27a-3p mimic and oe-ARHGAP25. ARHGAP25 was identified as an inhibitor of Wnt/β-catenin signaling, which has been reported to play a non-negligible role in BBB maintenance. Hence, miR-27a-3p may protect against BBB injury via ARHGAP2/Wnt/β-catenin axis. Western-blot unveiled that the expression of Wnt3a and β-catenin was significantly repressed by hemin treatment. Conversely, the protein level of Wnt3a and β-catenin was elevated in bEnd.3 cells transfected with miR-27a-3p mimic, while the increase was reversed in cells co-transfected with miR-27a-3p mimic + oe-ARHGAP25. Collectively, these data indicated that miR-27a-3p promoted endothelial barrier recovery through ARHGAP2/Wnt/β-catenin signaling pathway in in vitro ICH model.

## Discussion

To the best of our knowledge, this is the first work to document the role of OGD-stimulated ADEVs in BBB repair after ICH in vitro and in vivo. The significant findings of the current study are as follows: (1) OGD-ADEVs alleviated the neurological deficits and attenuated BBB disruption in mice ICH model; (2) OGD-ADEVs restored TEER and rescued TJPs in hemin-treated monolayer bEnd.3 cell model; (3) OGD-ADEVs were enriched with miRNA-27a-3p, which can be transferred into bEnd.3 cells; (4) miR-27a-3p effectively suppressed TJPs degradation and mitigated BBB injury via ARHGAP25/ Wnt/β-catenin signaling pathway.

We initially demonstrated in vitro the protective effect of C.M. from astrocyte (normal astrocyte and OGD-stimulated astrocyte) on barrier integrity in ICH cell model. It is now accepted that degrading TJPs, such as claudin-5, occludin and ZO-1, facilitates capillary leakage and contributes to the loss of BBB function [28, 29]. Our in vitro experiments revealed that hemin caused degradation of the occludin, claudin-5 and ZO-1 in bEnd.3 cells. These changes were reversed by C.M. from astrocytes. Importantly, C.M. from OGD-pretreated astrocytes was more effective than C.M. from normal astrocytes. GW4869 counteracted the capacity of C.M. from astrocytes of rescuing TJPs. The results indicated that ADEV, as a new paracrine mediator, plays an important role in intercellular communication between astrocytes and bEnd. 3 cells.

EVs are diverse, nano-sized vesicles secreted by virtually all cell types under both.

normal and pathological conditions, which deliver active molecules between cells locally or over longer distances [30, 31]. Recent studies have highlighted the centrality of ADEVs for the cell-to-cell communication in neuron-glia network [32, 33]. Several studies have suggested that ADEVs play an essential role in the physiological and pathological processes of the CNS [34, 35]. Similar to EVs from other sources, ADEVs also express markers, such as CD9, CD63, TSG101, and syntenin 1. The TEM and NTA results were consistent with those of EVs from other sources. ADEVs have been identified as a novel therapeutic strategy in acute neural injuries such as ischemic stroke and TBI [36, 37]. Effective suppression of neuroinflammation and neural injury has been achieved via the ADEVs administration. However, very little research has been conducted on the role of ADEVs in ICH. Our data demonstrated for the first time that ADEVs treatment lightened neurological deficits and reduced BBB disruption in the ICH mice model. Consistently, ADEVs significantly reduced BBB permeability and rescued TJPs degradation in vitro. Specifically, compared with N-ADEVs, OGD-ADEVs has more profound effect on mitigating BBB injury during ICH.

Increasing evidence suggests the cargo of EVs is highly dynamic and could be altered by the state and origin of the EV-secreting cell [38]. Using high-throughput miRNA sequencing, we demonstrated a unique miRNA signature in EVs from OGD-pretreated astrocytes. Among top 10 upregulated miRNA in OGD-ADEVs, miR‐27a‐3p retained our attention. Previously, an miRNA array revealed that miR-27a-3p was one of the down-regulated miRNAs in the serum of ICH patients [39], suggesting that it may have a pathogenic role in ICH. A study by Xi et al. showed that miR-27a-3p protected BBB integrity in mice with ICH [40]. In our study, qRT-PCR validated that miR-27a-3p showed a distinct difference between OGD-ADEVs and N-ADEVs. Hence, we prioritized miR-27a-3p for mechanistic investigation. When miR-27a-3p expression in OGD-ADEVs was inhibited, the ability of OGD-ADEVs to repress TJPs degradation was markedly compromised, suggesting the vital role of miR-27a-3p in ADEVs during this process. Subsequently, our findings indicated that miR-27a-3p in bEnd.3 cells was upregulated following ADEVs co-culturing, and this effect was abolished by ADEVs transfected with miR-27a-3p inhibitor. Co-localization of Cy3- miR-27a-3p mimic with PKH27-ADEVs further indicates that miR-27a-3p can be transferred from to hypoxic astrocytes cells via the route of EVs. Furthermore, bEnd.3 cells were transfected with miR-27a-3p mimic and inhibitor for the gain- and loss of-function investigation. Transfected with exogenous miR-27a-3p mimic to bEnd.3 cells exhibited protective effect against hemin-induced BBB disruption and TJPs reduction, whereas transfection with exogenous miR-27a-3p inhibitor significantly attenuated this effect. Collectively, ADEVs-mediated shuttling of miR-27a-3p into bEnd.3 cells may be sufficient to protect BBB from hemin injury.

In the subsequent experiment, target prediction of miR-27a-3p was performed using the miRDB, PITA and miRbase databases. ARHGAP25 was identified as a novel target of miR-27a-3p, and such targeting was further verified by subsequent luciferase reporter assays, and western blot analysis. ARHGAP25, a member of the ARHGAP family, is a GTPase-activating protein for Rac [41]. Evidence suggests that overexpression of ARHGAP17 exerts anticancer effect on colorectal cancer by inhibiting Wnt/β-catenin signaling pathway [42]. Previous publications have revealed the essential role of endothelial Wnt/β-catenin signaling in BBB formation and maintenance in healthy CNS [43], as well as in BBB repair in neurologic diseases [44]. Chen et al. revealed that immunoproteasome-activated Wnt/β-catenin signalling pathway reversed ischemia/hypoxia-induced BBB injury [45]. Wang et al. showed that BBB malfunction was restored via activating Wnt/β-catenin pathway in the AD mouse model [46]. In our studies, ARHGAP25 overexpression partially reversed the effect of miR-27a-3p on BBB integrity in ICH cell model. Hemin-induced reduction of wnt3a and β-catenin was attenuated by miR-27a-3p mimic, while ARHGAP25 overexpression suppressed miR-27a-3p-mediated elevation of Wnt3a and β-catenin. Together, our results demonstrated that miR-27a-3p mitigated BBB injury after ICH via targeting ARHGAP25 and regulating Wnt/β-catenin pathway.

## Conclusion

In summary, the findings in the present study highlight the important role of OGD-ADEVs in BBB repair during ICH. The mechanism of this effect is, at least in part, related to miR-27a-3p transfer from OGD-ADEVs to endothelial cells. ARHGAP25 may serve as a target gene to mediate the protective effect of miR-27a-3p via regulating the Wnt/β-catenin signaling pathway. Regulation of miR-27a-3p expression in OGD-ADEVs might be a promising strategy for BBB protection in patients with ICH.

### Supplementary Information

**Additional file 1:** **Figure S1.**Extracellular vesicles released by astrocytes restored the decrease of TJPs in bEnd.3 cells. A. Quantification of immunoblot of occludin, B. Quantification of immunoblot of Claudin-5, and C. Quantification of immunoblot of ZO-1. All data were expressed as mean ± SEM of at least 3 independent experiments. *P < 0.05, **P < 0.01. **Figure S2.**ADEVs treatment ameliorated neurological deficits and reduced brain water content after ICH. A. mNSS score at days 1, 3, and 7 in the sham, ICH, ICH+ N-ADEVs and ICH+ OGD-ADEVs groups. B. Brain water content of ipsilateral and contralateral hemisphere 24h after ICH. All data were expressed as mean ± SEM of at least 3 independent experiments. *P < 0.05, **P < 0.01**Figure S3.**ADEVs reversed the downregulated expression of TJPs after ICH. A. Quantification of immunoblot of occludin, B. Quantification of immunoblot of Claudin-5, and C. Quantification of immunoblot of ZO-1. All data were expressed as mean ± SEM of at least 3 independent experiments. *P < 0.05, **P < 0.01**Figure S4.**OGD-ADEVs miR-27a-3p attenuated the decrease in TJP levels. A. Quantification of immunoblot of occludin, B. Quantification of immunoblot of Claudin-5, and C. Quantification of immunoblot of ZO-1. All data were expressed as mean ± SEM of at least 3 independent experiments. *P < 0.05, **P < 0.01.

## Data Availability

The datasets analysed during the current study are available from the corresponding author on reasonable request.

## Abbreviations

ADEVs: Astrocyte-derived extracellular vesicles
ARHGAP25: Rho GTPase activating protein 25
BBB: Blood brain barrier
CM: Conditioned medium
EVs: Extracellular vesicles
ICH: Intracerebral hemorrhage
NTA: Nanosight tracking analysis
OGD: Oxygen glucose deprivation
TEER: Transendothelial electrical resistance
TEM: Transmission electron microscope
TJPs: Tight junction proteins

## References

[1] Al-Shahi Salman R, Frantzias J, Lee RJ, Lyden PD, Battey TWK, Ayres AM, Goldstein JN, Mayer SA, Steiner T, Wang X, Arima H, Hasegawa H, Oishi M, Godoy DA, Masotti L, Dowlatshahi D, Rodriguez-Luna D, Molina CA, Jang DK, Davalos A, Castillo J, Yao X, Claassen J, Volbers B, Kazui S, Okada Y, Fujimoto S, Toyoda K, Li Q, Khoury J, Delgado P, Sabín J, Hernández-Guillamon M, Prats-Sánchez L, Cai C, Kate MP, McCourt R, Venkatasubramanian C, Diringer MN, Ikeda Y, Worthmann H, Ziai WC, d'Esterre CD, Aviv RI, Raab P, Murai Y, Zazulia AR, Butcher KS, Seyedsaadat SM, Grotta JC, Martí-Fàbregas J, Montaner J, Broderick J, Yamamoto H, Staykov D, Connolly ES, Selim M, Leira R, Moon BH, Demchuk AM, Di Napoli M, Fujii Y, Anderson CS, Rosand J (2018). Absolute risk and predictors of the growth of acute spontaneous intracerebral haemorrhage: a systematic review and meta-analysis of individual patient data. Lancet Neurol.

[2] van Asch CJ, Luitse MJ, Rinkel GJ, van der Tweel I, Algra A, Klijn CJ (2010). Incidence, case fatality, and functional outcome of intracerebral haemorrhage over time, according to age, sex, and ethnic origin: a systematic review and meta-analysis, The Lancet. Neurology.

[3] Zahuranec DB, Lisabeth LD, Sánchez BN, Smith MA, Brown DL, Garcia NM, Skolarus LE, Meurer WJ, Burke JF, Adelman EE, Morgenstern LB (2014). Intracerebral hemorrhage mortality is not changing despite declining incidence. Neurology.

[4] Wu X, Luo J, Liu H, Cui W, Guo K, Zhao L, Bai H, Guo W, Guo H, Feng D, Qu Y (2020). Recombinant adiponectin peptide ameliorates brain injury following intracerebral hemorrhage by suppressing astrocyte-derived inflammation via the inhibition of Drp1-mediated mitochondrial fission. Transl Stroke Res.

[5] Keep RF, Andjelkovic AV, Xiang J, Stamatovic SM, Antonetti DA, Hua Y, Xi G (2018). Brain endothelial cell junctions after cerebral hemorrhage: changes, mechanisms and therapeutic targets. J Cerebral Blood Flow Metab Off J Int Soc Cerebral Blood Flow Metab.

[6] Lu T, Wang Z, Prativa S, Xu Y, Wang T, Zhang Y, Yu L, Xu N, Tang J, You W, Chen G, Zhang JH (2019). Macrophage stimulating protein preserves blood brain barrier integrity after intracerebral hemorrhage through recepteur d'origine nantais dependent GAB1/Src/β-catenin pathway activation in a mouse model. J Neurochem.

[7] Ronaldson PT, Davis TP (2020). Regulation of blood-brain barrier integrity by microglia in health and disease: a therapeutic opportunity. J Cerebral Blood Flow Metab Off J Int Soc Cerebral Blood Flow Metab.

[8] Harada K, Kamiya T, Tsuboi T (2015). Gliotransmitter release from astrocytes: functional developmental, and pathological implications in the brain. Front Neurosci.

[9] Jha MK, Kim JH, Song GJ, Lee WH, Lee IK, Lee HW, An SSA, Kim S, Suk K (2018). Functional dissection of astrocyte-secreted proteins: Implications in brain health and diseases. Prog Neurobiol.

[10] Finsterwald C, Magistretti PJ, Lengacher S (2015). Astrocytes: new targets for the treatment of neurodegenerative diseases. Curr Pharm Des.

[11] Pascua-Maestro R, González E, Lillo C, Ganfornina MD, Falcón-Pérez JM, Sanchez D (2018). Extracellular vesicles secreted by astroglial cells transport apolipoprotein D to neurons and mediate neuronal survival upon oxidative stress. Front Cell Neurosci.

[12] Upadhya R, Zingg W, Shetty S, Shetty AK (2020). Astrocyte-derived extracellular vesicles: Neuroreparative properties and role in the pathogenesis of neurodegenerative disorders. J Controll Release Off J Controll Release Soc.

[13] Luarte A, Henzi R, Fernández A, Gaete D, Cisternas P, Pizarro M, Batiz LF, Villalobos I, Masalleras M, Vergara R, Varas-Godoy M, Abarzua-Catalan L, Herrera-Molina R, Lafourcade C, Wyneken U (2020). Astrocyte-derived small extracellular vesicles regulate dendritic complexity through miR-26a-5p activity. Cells.

[14] Zhao S, Sheng S, Wang Y, Ding L, Xu X, Xia X, Zheng JC (2021). Astrocyte-derived extracellular vesicles: a double-edged sword in central nervous system disorders. Neurosci Biobehav Rev.

[15] Rouillard ME, Sutter PA, Durham OR, Willis CM, Crocker SJ (2021). Astrocyte-derived extracellular vesicles (ADEVs): deciphering their influences in aging. Aging Dis.

[16] Michinaga S, Koyama Y (2019). Dual roles of astrocyte-derived factors in regulation of blood-brain barrier function after brain damage. Int J Mol Sci.

[17] Spampinato SF, Bortolotto V, Canonico PL, Sortino MA, Grilli M (2019). Astrocyte-derived paracrine signals: relevance for neurogenic niche regulation and blood-brain barrier integrity. Front Pharmacol.

[18] Kriaučiūnaitė K, Kaušylė A, Pajarskienė J, Tunaitis V, Lim D, Verkhratsky A, Pivoriūnas A (2021). Immortalised hippocampal astrocytes from 3xTG-AD mice fail to support BBB integrity in vitro: role of extracellular vesicles in glial-endothelial communication. Cell Mol Neurobiol.

[19] Taylor X, Cisternas P, You Y, You Y, Xiang S, Marambio Y, Zhang J, Vidal R, Lasagna-Reeves CA (2020). A1 reactive astrocytes and a loss of TREM2 are associated with an early stage of pathology in a mouse model of cerebral amyloid angiopathy. J Neuroinflammation.

[20] Karuppagounder SS, Alim I, Khim SJ, Bourassa MW, Sleiman SF, John R, Thinnes CC, Yeh TL, Demetriades M, Neitemeier S, Cruz D, Gazaryan I, Killilea DW, Morgenstern L, Xi G, Keep RF, Schallert T, Tappero RV, Zhong J, Cho S, Maxfield FR, Holman TR, Culmsee C, Fong GH, Su Y, Ming GL, Song H, Cave JW, Schofield CJ, Colbourne F, Coppola G, Ratan RR (2016). Therapeutic targeting of oxygen-sensing prolyl hydroxylases abrogates ATF4-dependent neuronal death and improves outcomes after brain hemorrhage in several rodent models. Sci Transl Med.

[21] Hu G, Yao H, Chaudhuri AD, Duan M, Yelamanchili SV, Wen H, Cheney PD, Fox HS, Buch S (2012). Exosome-mediated shuttling of microRNA-29 regulates HIV Tat and morphine-mediated neuronal dysfunction. Cell Death Dis.

[22] Ding H, Jia Y, Lv H, Chang W, Liu F, Wang D (2021). Extracellular vesicles derived from bone marrow mesenchymal stem cells alleviate neuroinflammation after diabetic intracerebral hemorrhage via the miR-183-5p/PDCD4/NLRP3 pathway. J Endocrinol Invest.

[23] Pei X, Li Y, Zhu L, Zhou Z (2019). Astrocyte-derived exosomes suppress autophagy and ameliorate neuronal damage in experimental ischemic stroke. Exp Cell Res.

[24] Huang J, Ge S, Luo D, Du R, Wang Y, Liu W, Wang G, Yin T (2022). The endothelium permeability after bioresorbable scaffolds implantation caused by the heterogeneous expression of tight junction proteins. Mater Today Bio.

[25] Ma F, Zhang X, Yin KJ (2020). MicroRNAs in central nervous system diseases: a prospective role in regulating blood-brain barrier integrity. Exp Neurol.

[26] Sun P, Liu DZ, Jickling GC, Sharp FR, Yin KJ (2018). MicroRNA-based therapeutics in central nervous system injuries. J Cerebral Blood Flow Metab Off J Int Soc Cerebral Blood Flow Metabol.

[27] Hammad S, Mabondzo A, Hamoudi R, Harati R (2022). Regulation of P-glycoprotein by miR-27a-3p at the brain endothelial barrier. J Pharm Sci.

[28] Winkler L, Blasig R, Breitkreuz-Korff O, Berndt P, Dithmer S, Helms HC, Puchkov D, Devraj K, Kaya M, Qin Z, Liebner S, Wolburg H, Andjelkovic AV, Rex A, Blasig IE, Haseloff RF (2021). Tight junctions in the blood-brain barrier promote edema formation and infarct size in stroke—ambivalent effects of sealing proteins. J Cerebral Blood Flow Metab Off J Int Soc Cerebral Blood Flow Metab.

[29] Li Y, Zhu ZY, Huang TT, Zhou YX, Wang X, Yang LQ, Chen ZA, Yu WF, Li PY (2018). The peripheral immune response after stroke-A double edge sword for blood-brain barrier integrity. CNS Neurosci Ther.

[30] Fitzner D, Schnaars M, van Rossum D, Krishnamoorthy G, Dibaj P, Bakhti M, Regen T, Hanisch UK, Simons M (2011). Selective transfer of exosomes from oligodendrocytes to microglia by macropinocytosis. J Cell Sci.

[31] Ibrahim SA, Khan YS (2022). Histology extracellular vesicles in StatPearls StatPearls publishing copyright © 2022.

[32] Global, regional, and national burden of stroke and its risk factors 1990–2019 (2019). A systematic analysis for the global burden of disease study. Lancet Neurol.

[33] Patel MR, Weaver AM (2021). Astrocyte-derived small extracellular vesicles promote synapse formation via fibulin-2-mediated TGF-β signaling. Cell Rep.

[34] Peng D, Wang Y, Xiao Y, Peng M, Mai W, Hu B, Jia Y, Chen H, Yang Y, Xiang Q, Su Z, Zhang Q, Huang Y (2022). Extracellular vesicles derived from astrocyte-treated with haFGF(14–154) attenuate Alzheimer phenotype in AD mice. Theranostics.

[35] Pistono C, Bister N, Stanová I, Malm T (2020). Glia-Derived extracellular vesicles: role in central nervous system communication in health and disease. Front Cell Develop Biol.

[36] Chen CY, Chao YM, Lin HF, Chen CJ, Chen CS, Yang JL, Chan JYH, Juo SH (2020). miR-195 reduces age-related blood-brain barrier leakage caused by thrombospondin-1-mediated selective autophagy. Aging Cell.

[37] Xu L, Cao H, Xie Y, Zhang Y, Du M, Xu X, Ye R, Liu X (2019). Exosome-shuttled miR-92b-3p from ischemic preconditioned astrocytes protects neurons against oxygen and glucose deprivation. Brain Res.

[38] de Jong OG, Verhaar MC, Chen Y, Vader P, Gremmels H, Posthuma G, Schiffelers RM, Gucek M, van Balkom BW (2012). Cellular stress conditions are reflected in the protein and RNA content of endothelial cell-derived exosomes. J Extracellular Vesicles.

[39] Zhu Y, Wang JL, He ZY, Jin F, Tang L (2015). Association of altered serum microRNAs with perihematomal edema after acute intracerebral hemorrhage. PLoS ONE.

[40] Xi T, Jin F, Zhu Y, Wang J, Tang L, Wang Y, Liebeskind DS, Scalzo F, He Z (2018). miR-27a-3p protects against blood-brain barrier disruption and brain injury after intracerebral hemorrhage by targeting endothelial aquaporin-11. J Biol Chem.

[41] Huang WK, Chen Y, Su H, Chen TY, Gao J, Liu Y, Yeh CN, Li S (2021). ARHGAP25 inhibits pancreatic adenocarcinoma growth by suppressing glycolysis via AKT/mTOR pathway. Int J Biol Sci.

[42] Tao L, Zhu Y, Gu Y, Zheng J, Yang J (2019). ARHGAP25: a negative regulator of colorectal cancer (CRC) metastasis via the Wnt/β-catenin pathway. Eur J Pharmacol.

[43] Stenman JM, Rajagopal J, Carroll TJ, Ishibashi M, McMahon J, McMahon AP (2008). Canonical Wnt signaling regulates organ-specific assembly and differentiation of CNS vasculature. Science.

[44] Liebner S, Corada M, Bangsow T, Babbage J, Taddei A, Czupalla CJ, Reis M, Felici A, Wolburg H, Fruttiger M, Taketo MM, von Melchner H, Plate KH, Gerhardt H, Dejana E (2008). Wnt/beta-catenin signaling controls development of the blood-brain barrier. J Cell Biol.

[45] Chen XY, Wan SF, Yao NN, Lin ZJ, Mao YG, Yu XH, Wang YZ (2021). Inhibition of the immunoproteasome LMP2 ameliorates ischemia/hypoxia-induced blood-brain barrier injury through the Wnt/β-catenin signalling pathway. Mil Med Res.

[46] Wang Q, Huang X, Su Y, Yin G, Wang S, Yu B, Li H, Qi J, Chen H, Zeng W, Zhang K, Verkhratsky A, Niu J, Yi C (2022). Activation of Wnt/β-catenin pathway mitigates blood-brain barrier dysfunction in Alzheimer's disease. Brain J Neurol.

