# Supplementary figures and images for: Oxygen glucose deprivation-pretreated astrocyte-derived exosomes attenuates intracerebral hemorrhage (ICH)-induced BBB disruption through miR-27a-3p /ARHGAP25/Wnt/β-catenin axis
Source: Fluids Barriers CNS. 2024 Jan 19;21:8. doi: 10.1186/s12987-024-00510-2 (PMC10799414; doi:10.1186/s12987-024-00510-2)

Figure S1


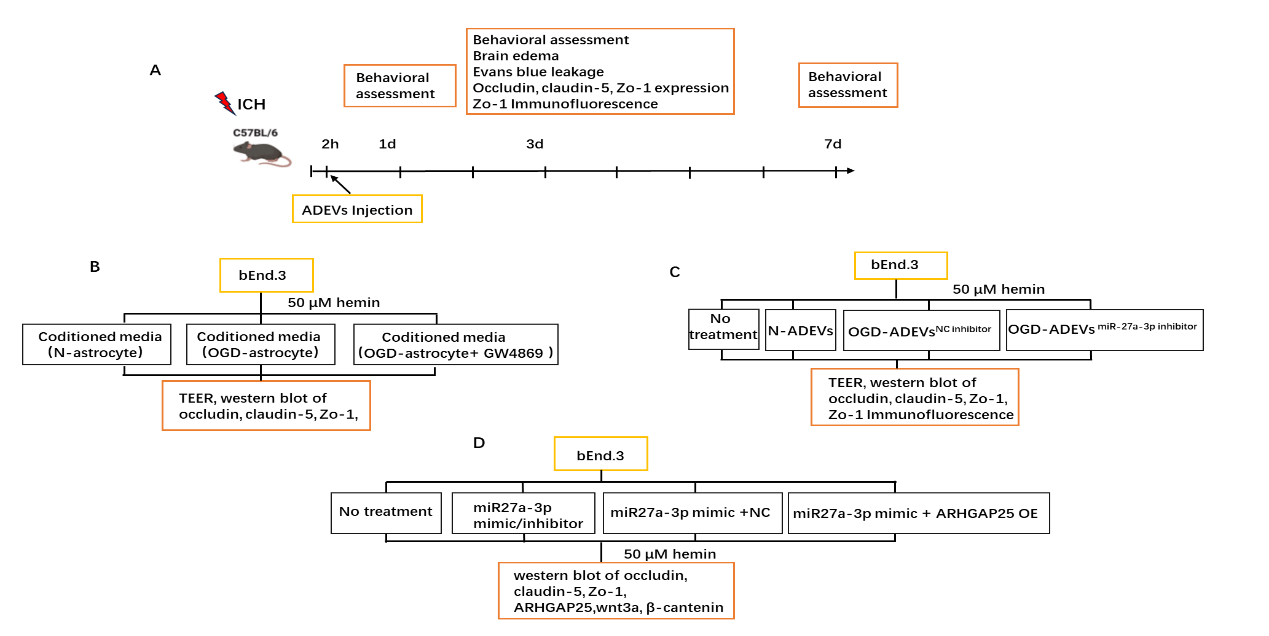


Figure S2


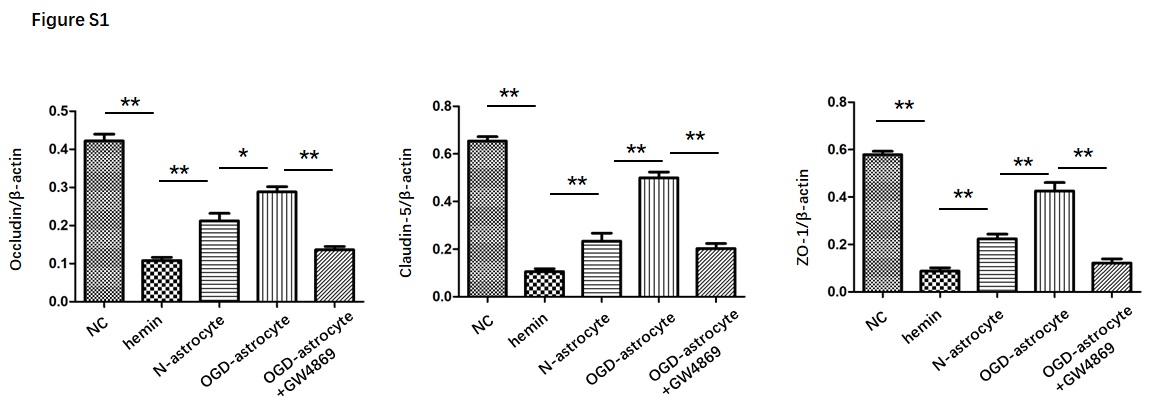


Figure S3


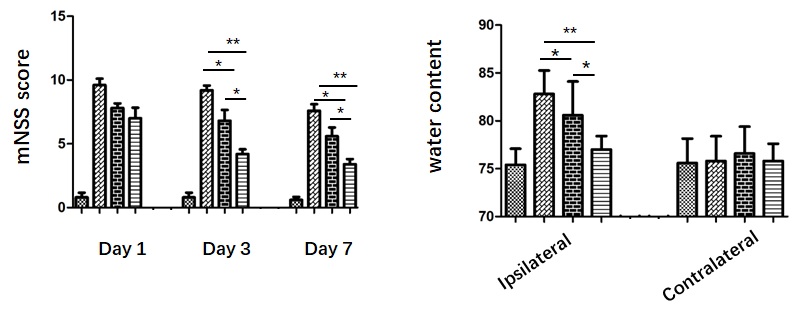


Figure S4


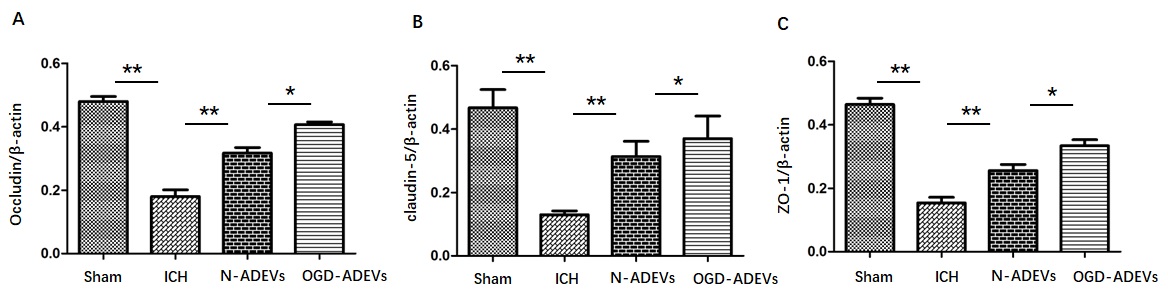


Figure S5


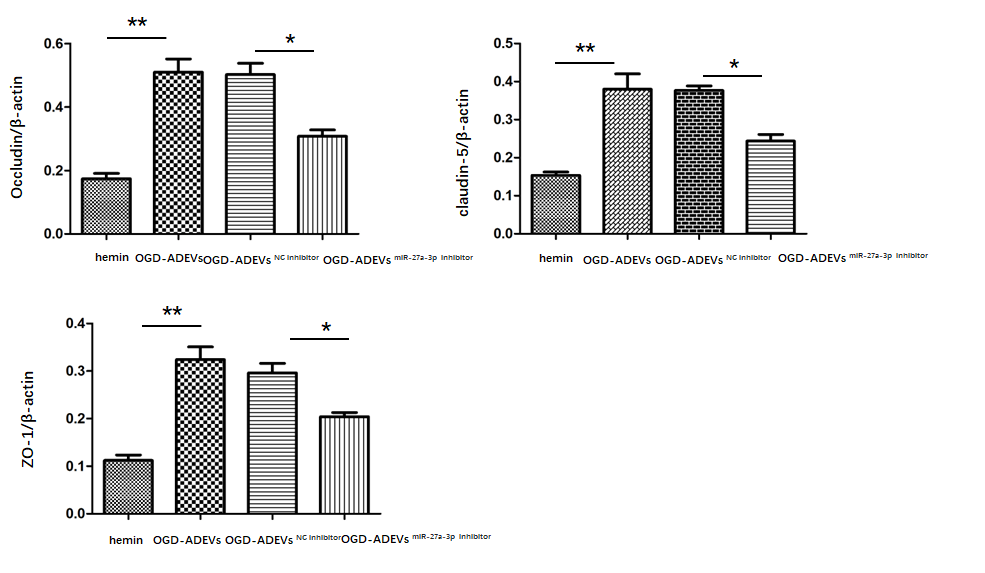

Supplement: Supplementary file 1 — Additional file 1: Figure S1.Extracellular vesicles released by astrocytes restored the decrease of TJPs in bEnd.3 cells. A. Quantification of immunoblot of occludin, B. Quantification of immunoblot of Claudin-5, and C. Quantification of immunoblot of ZO-1. All data were expressed as mean ± SEM of at least 3 independent experiments. *P < 0.05, **P < 0.01. Figure S2.ADEVs treatment ameliorated neurological deficits and reduced brain water content after ICH. A. mNSS score at days 1, 3, and 7 in the sham, ICH, ICH+ N-ADEVs and ICH+ OGD-ADEVs groups. B. Brain water content of ipsilateral and contralateral hemisphere 24h after ICH. All data were expressed as mean ± SEM of at least 3 independent experiments. *P < 0.05, **P < 0.01Figure S3.ADEVs reversed the downregulated expression of TJPs after ICH. A. Quantification of immunoblot of occludin, B. Quantification of immunoblot of Claudin-5, and C. Quantification of immunoblot of ZO-1. All data were expressed as mean ± SEM of at least 3 independent experiments. *P < 0.05, **P < 0.01Figure S4.OGD-ADEVs miR-27a-3p attenuated the decrease in TJP levels. A. Quantification of immunoblot of occludin, B. Quantification of immunoblot of Claudin-5, and C. Quantification of immunoblot of ZO-1. All data were expressed as mean ± SEM of at least 3 independent experiments. *P < 0.05, **P < 0.01. [file 12987_2024_510_MOESM1_ESM.docx]
